# miRNA-Based Signature Associated With Tumor Mutational Burden in Colon Adenocarcinoma

**DOI:** 10.3389/fonc.2021.634841

**Published:** 2021-06-23

**Authors:** Weijie Xue, Yixiu Wang, Yuwei Xie, Chenyu Yang, Zhiqi Gong, Chunyang Guan, Chuqing Wei, Chengzhan Zhu, Zhaojian Niu

**Affiliations:** ^1^ Department of Gastrointestinal Surgery, The Affiliated Hospital of Qingdao University, Qingdao, China; ^2^ Department of Hepatic Surgery, Shanghai Cancer Center, Shanghai Medical College, Fudan University, Shanghai, China; ^3^ Department of Hepatobiliary and Pancreatic Surgery, The Affiliated Hospital of Qingdao University, Qingdao, China; ^4^ Shandong Key Laboratory of Digital Medicine and Computer Assisted Surgery, The Affiliated Hospital of Qingdao University, Qingdao, China; ^5^ Interventional Operating Room, The Affiliated Hospital of Qingdao University, Qingdao, China; ^6^ Shandong University Affiliated Shandong Tumor Hospital and Institute, Jinan, China

**Keywords:** colon adenocarcinoma, bioinformatics, tumor mutational burden, immunology, signature

## Abstract

Colon adenocarcinoma (COAD) is one of the most common malignant tumors. Tumor mutation burden (TMB) has become an independent biomarker for predicting the response to immune checkpoint inhibitors (ICIs). miRNAs play an important role in cancer-related immune regulation. However, the relationship between miRNA expression and TMB in COAD remains unclear. Therefore, the transcriptome profiling data, clinical data, mutation annotation data, and miRNA expression profiles for cases of COAD were downloaded from the TCGA database. Subsequently, 323 COAD cases were randomly divided into training and test sets. The differential expression of miRNAs in the high and low TMB groups in the training set was obtained as a signature using the least absolute shrinkage and selection operator (LASSO) logistic regression and verified in the test set. Based on the LASSO method, principal component analysis (PCA), and ROC, we found that the signature was credible because it can discriminate between high and low TMB levels. In addition, the correlation between the 18-miRNA-based signature and immune checkpoints was performed, followed by qRT-PCR, to measure the relative expression of 18 miRNAs in COAD patients. The miRNA-based model had a strong positive correlation with TMB and a weak positive correlation with CTLA4 and CD274 (PD-L1). However, no correlation was observed between the model and SNCA (PD-1). Finally, enrichment analysis of the 18 miRNAs was performed to explore their biological functions. The results demonstrated that 18 miRNAs were involved in the process of immunity and cancer pathways. In conclusion, the 18-miRNA-based signature can effectively predict and discriminate between the different TMB levels of COAD and provide a guide for its treatment with ICIs.

## Introduction

Colon adenocarcinoma (COAD) is a common malignant neoplasm of the digestive system. It has become the third most common cancer worldwide and the second leading cause of cancer-related mortality (1). Patients usually present with diarrhea, abdominal pain, and bloody stool, which develop during the middle and late stages of the disease. The quality of life of patients is often low, and most have a poor prognosis. In recent years, although great progress has been made in the treatment of COAD with surgery, chemotherapy, and targeted therapy, the five-year survival rate of patients with advanced COAD remains low. After nearly a hundred years of effort, tumor immunotherapy has become one of the means to treat tumors (2). Programmed death protein-1 (PD-1)/programmed death receptor ligand (PD-L1) and immune checkpoint inhibitors (ICIs) have been approved by the Food and Drug Administration (FDA) for the treatment of non-small cell lung cancer, melanoma, and head and neck squamous cell carcinoma (3), etc. In recent years, PD-1/PD-L1 ICIs have achieved encouraging results in the treatment of advanced COAD (4–6). The expression of PD-L1 can be used as a biomarker of PD-1/PD-L1 inhibitor therapy to help predict the response to treatment. However, less than 30% of patients exhibit a long-term response to immune checkpoint treatments (7, 8). This suggests that the expression of PD-L1 is probably not a unique identifying responder, and it is therefore necessary to identify better responders.

Tumor mutational burden (TMB) is a potentially effective biomarker for predicting the survival of cancer patients treated with ICIs (9–11). In addition, TMB expression is independent of PD-L1 expression. TMB is defined as the total number of somatic gene coding errors, and base substitution, gene insertion, or deletion errors detected per million bases (MB) (12, 13). Currently, next generation sequencing (NGS) is the most commonly used method for detecting TMB. However, there are some challenges to using this method. For example, the high cost, the need for large amounts of tumor DNA, and the use of different platforms, which lead to inconsistent results (14). A high TMB level may result in the modification of a protein encoded by a mutant gene. This protein is recognized by the immune system as “non-self” and this activates a specific anti-tumor immune response as a new tumor-specific antigen, increasing the probability of these tumor cells being killed (15).

microRNAs (miRNAs) play an important role in the post-transcriptional regulation of translation of mutant genes into modified proteins. miRNAs are classes of non-coding single-stranded RNA molecules encoded by endogenous genes with a length of approximately 22 nucleotides. They are involved in the regulation of post-transcriptional gene expression in animals and plants (16). Abnormal expression of miRNAs is often associated with many diseases, especially cancer (17, 18). In recent years, with the increase in research on miRNAs, we have realized that they play an irreplaceable role in cancer and several studies have shown that miRNAs may serve as prognostic markers for various cancers (18). miRNAs play an important role in the tumor microenvironment (19–22). Studies have shown that the expression of miRNAs in some tumors is highly specific, and that it plays a vital role in the immune response, especially in early regulation. In the process of tumor development, the immune system plays a key role, and there is a close interaction between tumor cells and immune cells through the release of a variety of signals (23, 24). It has been shown that miRNAs can be used as a medium for communication between tumor cells and immune cells. Therefore, we predicted that the expression of miRNAs correlates with TMB levels, and that miRNAs can be used as molecular markers for predicting TMB levels. To verify our predictions, the mutation annotation data and miRNA expression profiles of patients with COAD were downloaded from the Cancer Genome Atlas (TCGA, https://gdc.cancer.gov/) and used to establish an miRNA-based model to predict TMB levels.

## Materials and Methods

### Data Collection and Processing

The transcriptome profiling data, clinical data, mutation annotation data, and miRNA expression profiles of patients with COAD were obtained from the TCGA database. The data were processed according to PERL. This included integration and normalization of the transcriptome profiling data, the extraction of clinical information, the merging of the miRNA data, and counting of TMB levels of the mutation annotation data. A threshold of 10 was used, a TMB count greater than or equal to 10 was considered high, and a count less than 10 was considered low. The TMB and miRNA expression data were merged using the “limma” package in R software (25). As a result, a total of 323 tumor samples common to the mutation annotation data and miRNA data were identified. The 323 tumor samples were stochastically divided into training (60%) and test (40%) sets using the “caret” package in R (26). The two sets of clinicopathological data were statistically analyzed.

### Extraction and Analysis of Differentially Expressed miRNAs

The differential expression of miRNAs in the high and low TMB groups in the training set was analyzed using the “limma” package in R. A fold change of FC >1.5, and P values <0.01, after adjustment by the false discovery rate, were used as the criteria for screening for statistical significance. In addition, a heat map was drawn by “pheatmap” in R to visualize the statistically significant differential expression of miRNAs in the high and low TMB groups.

### Least Absolute Shrinkage and Selection Operator (LASSO) and Principal Component Analysis (PCA) of Differentially Expressed miRNAs

Differentially expressed miRNAs were identified from the training set. The LASSO logistic regression model was developed using the “glmnet” package in R. The model was used to predict the optimum differential expression of miRNAs as a signature for discriminating between the two TMB levels (27). In addition, differentially expressed miRNAs were used to perform the PCA using the “ggplot” package in R. Finally, a PCA of the optimum differentially expressed miRNAs was used to validate the reliability of the LASSO logistic regression model (28).

### The Validation of the miRNA-Based Model for Predicting the TMB Level

The model obtained from the training set for predicting TMB levels was an miRNA-based signature discriminator. We introduced the model into the test set to verify the robustness and general applicability of the model. The discriminator index was created for each sample using the regression coefficients of the LASSO analysis to calculate the expression value of the optimum miRNAs using the following formula: index = β1*Exp_miRNA1_ + β2*Exp_miRNA2_ + β3*Exp_miRNA3_ + …++ βn*Exp_miRNAn_. “β” is the regression coefficient of miRNA, and “Exp” is the expression of the miRNAs. The efficiency of the model was evaluated by accuracy, sensitivity (SE), specificity (SP), positive predictive value (PPV), negative predictive value (NPV), and area under the receiver operating characteristic (ROC) curve. The ROC curves were created and analyzed by the “pROC” package in R (29).

### Correlation Analysis of the Model With TMB Levels and Immune Checkpoints

To assess the correlation between the model and TMB levels, the calculated index of each sample in the total set was used together with the integrated data of the TMB and miRNA using the “limma”, “ggplot2”, and “ggpubr” packages in R (30). The three immune checkpoints were PD-1(SNCA), PD-L1 (CD274), and CTLA-4. In addition, the calculated index of each sample in the total set was used together with the pre-processed transcriptome profiling data to analyze the correlation between the model and immune checkpoints.

### Enrichment Analysis for the Target Genes Prediction of the miRNAs in Model

The corresponding database files were downloaded from TargetScan (http://www.targetscan.org/vert_72/), miRTarBase (http://mirtarbase.mbc.nctu.edu.tw/php/index.php), and miRDB (http://mirdb.org/) in TSV format. miRNAs in the model were introduced into the three files to predict the target genes using PERL. The standard for determining the target genes of miRNAs was supported by all three databases. Using the “clusterprofiler” package in R (31), the biological processes and molecular functions of miRNAs were inferred by Gene Ontology (GO) analysis of the target genes. The Kyoto Encyclopedia of Genes and Genomes (KEGG) pathway enrichment analysis of target genes of the miRNAs in the model were performed and visualized using the “clusterProfiler” package in R.

### Real Time-PCR Analysis of the miRNAs

We collected tumors and corresponding non-tumor tissues from 30 patients of the Department of Gastrointestinal Surgery of the Affiliated Hospital of Qingdao University. Total RNA was extracted from 30 tumor and 30 non-tumor tissue samples *via* Vazyme #R701 (RNA-easy Isolation Reagent, Vazyme, Nanjing, China) in accordance with the manufacturer’s instructions. Subsequently, a reverse transcription reaction was performed using Vazyme.MR101-01 (miRNA 1st Strand cDNA Synthesis Kit, Nanjing, China). Quantitative real-time PCR (qRT-PCR) was performed using a LightCycler 480 (Roche, Basel, Switzerland) and Vazyme #MQ101 (miRNA Universal SYBR ^®^ qPCR Master Mix, Nanjing, China). The primer sequences of the miRNAs are listed in **Table 1**. The data were standardized using a control group of the small nuclear RNA U6.

**Table 1:** Sequence of each miRNA.

hsa-miR-296-5p	3’…UGUCCUAACUCCCCCCCGGGA
hsa-miR-155-5p	3’…UGGGGAUAGUGCUAAUCGUAAUU
hsa-miR-6761-5p	5’…UCUGAGAGAGCUCGAUGGCAG
hsa-miR-582-5p	5’…UUACAGUUGUUCAACCAGUUACU
hsa-miR-452-3p	5’…CUCAUCUGCAAAGAAGUAAGUG
hsa-miR-330-5p	5’…TCTCTGGGCCTGTGTCTTAGGC
hsa-miR-3127-5p	5’…AUCAGGGCUUGUGGAAUGGGAAG
hsa-miR-146b-5p	5’…UGAGAACUGAAUUCCAUAGGCU
hsa-miR-99a-5p	5’…AACCCGUAGAUCCGAUCUUGUG
hsa-miR-874-3p	5’…CUGCCCUGGCCCGAGGGACCGA
hsa-miR-132-3p	5’…UAACAGUCUACAGCCAUGGUCG
hsa-miR-625-3p	5’…GACUAUAGAACUUUCCCCCUCA
hsa-miR-552-5p	5’…GUUUAACCUUUUGCCUGUUGG
hsa-miR-195-3p	5’…CCAAUAUUGGCUGUGCUGCUCC
hsa-miR-452-5p	5’…AACUGUUUGCAGAGGAAACUGA
hsa-miR-224-5p	5’…CAAGUCACUAGUGGUUCCGUU
hsa-miR-582-3p	5’…UAACUGGUUGAACAACUGAACC
hsa-miR-592	5’…UUGUGUCAAUAUGCGAUGAUGU
U6	5’…GCTTCGGCAGCACATATACT

### Statistical Analysis

Statistical analyses of the clinical data were performed using the Chi-square test. The expression of the miRNAs in the samples in the high and low TMB groups was compared using the unpaired t-test. The qRT-PCR data were analyzed using the unpaired t test (GraphPad Prism 7). All statistical analyses were performed using R and GraphPad Prism.

## Results

### Establishment and Identification of Differentially Expressed miRNAs

**Figure 1:**
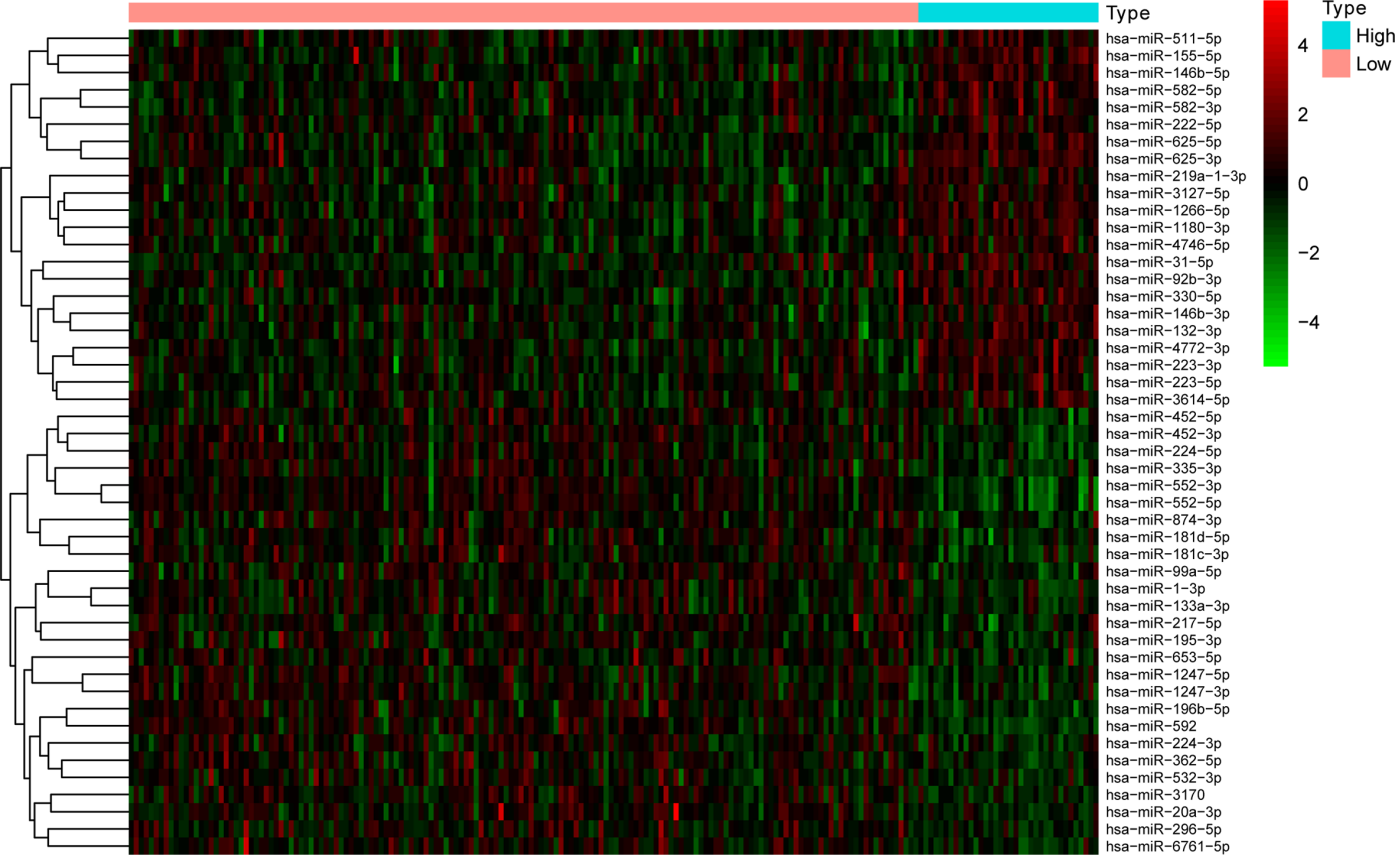
The 18 differentially expressed miRNAs can essentially identify COAD samples with high and low levels of TMB.

The clinicopathological data of patients in the training and test sets were analyzed, and no statistical significance was found between the two sets, as shown in **Table 2**. There were 36 and 158 samples in the high and low TMB groups, respectively. |log2FC| >0.263 and the P value adjusted by a false discovery rate <0.01, were used as screening criteria, and 48 miRNAs were found to be differentially expressed in samples with high and low TMB levels. There were 22 upregulated and 26 downregulated differentially expressed miRNAs in samples with high TMB levels. The visualization of miRNA expression in the high and low TMB groups is shown in **Figure 1**. The results of the heatmap demonstrate that these differentially expressed miRNAs can be used to identify samples with either high or low levels of TMB.

**Table 2:** Clinicopathology characteristics of patients with COAD.

Covariates		Training set	Test set	P-value
Age	<=65	81(41.75%)	57(44.19%)	0.7503
	>65	113(58.25%)	72(55.81%)	
Gender	FEMALE	95(48.97%)	61(47.29%)	0.8551
	MALE	99(51.03%)	68(52.71%)	
Stage	I-II	110(56.7%)	66(51.16%)	0.5004
	III-IV	79(40.72%)	57(44.19%)	
	unknow	5(2.58%)	6(4.65%)	
T	T1-2	34(17.53%)	30(23.26%)	0.2615
	T3-4	160(82.47%)	99(76.74%)	
M	M0	136(70.1%)	91(70.54%)	0.1279
	M1	37(19.07%)	14(10.85%)	
	unknow	21(10.82%)	24(18.6%)	
N	N0	116(59.79%)	72(55.81%)	0.5518
	N1-3	78(40.21%)	57(44.19%)	

### Signature Acquirement Through LASSO and Identification

**Figure 2:**
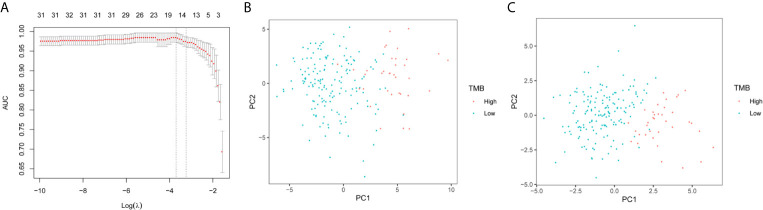
LASSO logistic regression model and PCA. **(A)** Selection of arguments in the LASSO method. **(B)** PCA of 49 differential expression miRNAs. **(C)** PCA of 18 differentially expressed miRNAs selected by the LASSO method.

The expression of 48 miRNAs in the training set was analyzed using LASSO logistic regression to establish a model based on miRNA as a discriminator of TMB levels. After LASSO analysis, 18 miRNAs with the largest area under the curve (AUC) were identified as a signature for discriminating between TMB levels, as shown in **Figure 2A**. These 18 miRNAs were hsa-miR-296-5p, hsa-miR-155-5p, hsa-miR-6761-5p, hsa-miR-582-5p, hsa-miR-452-3p, hsa-miR-330-5p, hsa-miR-3127-5p, hsa-miR-146b-5p, hsa-miR-99a-5p, hsa-miR-874-3p, hsa-miR-132-3p, hsa-miR-625-3p, hsa-miR-552-5p, hsa-miR-195-3p, hsa-miR-452-5p, hsa-miR-224-5p, hsa-miR-582-3p, and hsa-miR-592. **Figures 2B, C** show the results of the PCA of 49 differentially expressed miRNAs and 18 miRNAs in the model, respectively. It was confirmed that the 18 miRNAs identified by LASSO can effectively discriminate between samples with high or low TMB levels.

### The Validation of miRNA-based Model

According to the LASSO regression analysis, the formula is as follows:

$$index=\;\left(-0.0218449096244075*EXP_{hsa-miR-296-5p}\right)+\left(0.140980802896763*EXP_{hsa-miR-155-5p}\right)+\left(-0.0618130806762854*EXP_{hsa-miR-6761-5p}\right)+\left(0.792005574142893*EXP_{hsa-miR-582-5p}\right)+\left(0.0400501457312491*EXP_{hsa-miR-452-3p}\right)+\;\left(0.351535745471987*EXP_{hsa-miR-330-5p}\right)+\left(0.321466850321636*EXP_{hsa-miR-3127-5p}\right)+\left(0.306550028173435*EXP_{hsa-miR-146b-5p}\right)+\left(-0.0129214249555584*EXP_{hsa-miR-99a-5p}\right)+\left(-0.291557428450146*EXP_{hsa-miR-874-3p}\right)+\left(0.312801874996257*EXP_{hsa-miR-132-3p}\right)+\left(0.71353375237544*EXP_{hsa-miR-625-3p}\right)+\left(-0.339018514344295*EXP_{hsa-miR-552-5p}\right)+\left(-0.506312988185146*EXP_{hsa-miR-195-3p}\right)+\left(-0.203390691480266*EXP_{hsa-miR-452-5p}\right)+\left(-0.038165302905481*EXP_{hsa-miR-224-5p}\right)+\left(0.0188966224605559*EXP_{hsa-miR-582-3p}\right)+\left(-0.303904239862044*EXP_{hsa-miR-592}\right)$$

**Figure 3:**
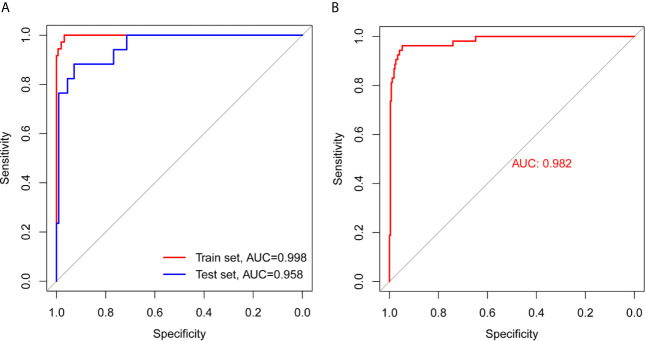
ROC curve analysis of training set, test set, and total set. **(A)** ROC curves of training set and test set. **(B)** ROC curve of total set.

The results of the analysis using the model are presented in **Table 3**. The accuracies in the training, test, and total sets were 0.9753, 0.964, and 0.9598, respectively. These results confirm that the model is highly reliable. The analysis of the ROC curve showed that the AUC of the training, test, and total set was 0.998, 0.958, and 0.982, respectively. These results indicated that there was no significant difference between the training and test sets, and verified the accuracy of the model (**Figures 3A, B**).

**Table 3:** Performance of 18-miRNA-based signature of TMB in COAD.

ID	SE	SP	PPV	NPV	Accuracy	AUC
Train	0.8889	1	1	0.9753	0.9794	0.9984
Test	0.7647	0.9554	0.7222	0.964	0.9302	0.958
Total	0.8491	0.9815	0.9	0.9707	0.9598	0.9817

### Correlation Analysis Between the Model and the TMB Level and Immune Checkpoints, Respectively

**Figure 4:**
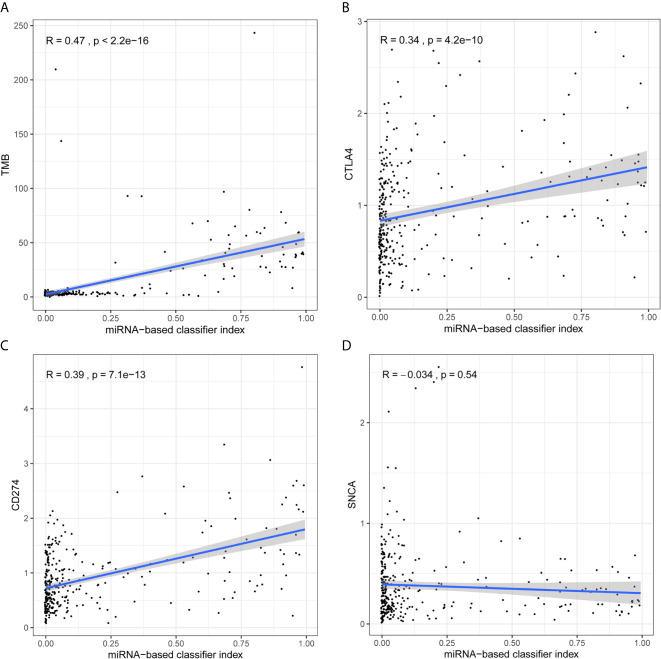
Analysis of correlation between signature and TMB levels and immune checkpoints. **(A)** The 18-miRNA-based signature has a strong positive correlation with TMB levels. **(B)** The 18-miRNA-based signature has a weak positive correlation with CTLA4. **(C)** The 18-miRNA-based signature has a weak positive correlation with CD274 (PDL-1). **(D)** The 18-miRNA-based signature has no correlation with SNCA (PD-1).

The following section analyzes the correlation between the model and TMB levels, and between the model and immune checkpoints. The index of each sample in the total set was calculated and combined with the integrated data of TMB and miRNA. In addition, the index was combined with pre-processed transcriptome profiling data. As demonstrated in **Figures 4A–D**, there was a strong positive correlation between the miRNA-based model and TMB levels (**Figure 4A**), and weak positive correlations with CTLA4 (**Figure 4B**) and CD274 (**Figure 4C**). However, there was no correlation between the model and SNCA (PD-1) (Pearson R = -0.034, *P* = 0.54, **Figure 4D**).

### Enrichment Analysis for Target Genes Prediction of miRNAs in the Model

**Figure 5:**
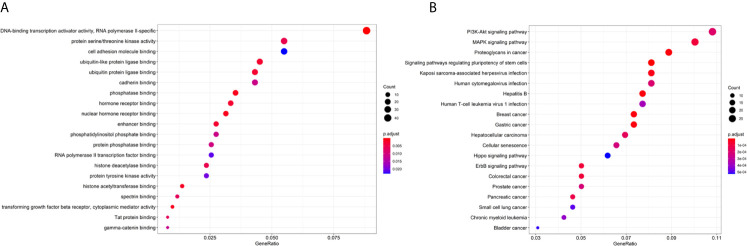
Enrichment analysis of the 18 miRNAs. **(A)** GO analysis of the target genes of 18 miRNAs. **(B)** KEGG analysis of the target genes of 18 miRNAs.

As shown in **Figures 5A, B**, target genes corresponding to the miRNAs were predicted from the three databases. GO contains three aspects of functional information: the biological process in which genes are involved, the location of the cells, and the function of the molecules. In the analysis, we found that the target genes were enriched in “DNA-binding transcription activator activity, RNA polymerase II-specific,” “transforming growth factor beta receptor, cytoplasmic mediator activity”, and “phosphatase binding”, etc. KEGG provides an understanding of advanced functions and biological systems at the molecular level. The results of the KEGG analysis showed that the target genes of 18 miRNAs were mainly enriched in cancer and cancer-related signaling pathways, for example, “Colorectal cancer” and the “MAPK signaling pathway.”

### Relative Expression of miRNAs in COAD

**Figure 6:**
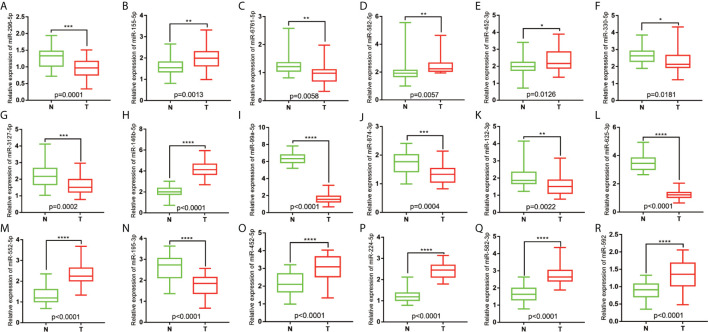
Relative expression of miRNAs in COAD patients. **(A)** The relative expression level of hsa-miR-296-5p in COAD. **(B)** The relative expression level of hsa-miR-155-5p in COAD. **(C)** The relative expression level of hsa-miR-6761-5p in COAD. **(D)** The relative expression level of hsa-miR-582-5p in COAD. **(E)** The relative expression level of hsa-miR-452-3p in COAD. **(F)** The relative expression level of hsa-miR-330-5p in COAD. **(G)** The relative expression level of hsa-miR-3127-5p in COAD. **(H)** The relative expression level of hsa-miR-146b-5p in COAD. **(I)** The relative expression level of hsa-miR-99a-5p in COAD. **(J)** The relative expression level of hsa-miR-874-3p in COAD. **(K)** The relative expression level of hsa-miR-132-3p in COAD. **(L)** The relative expression level of hsa-miR-625-3p in COAD. **(M)** The relative expression level of hsa-miR-552-5p in COAD. **(N)** The relative expression level of hsa-miR-195-3p in COAD. **(O)** The relative expression level of hsa-miR-452-5p in COAD. **(P)** The relative expression level of hsa-miR-224-5p in COAD. **(Q)** The relative expression level of hsa-miR-582-3p in COAD. **(R)** The relative expression level of and hsa-miR-592 in COAD. *P<0.05; **P<0.01; ***P<0.001; ****P<0.0001.

The tumor and non-tumor tissues of 30 patients with COAD were measured using qRT-PCR. The results showed that miR-296-5p, miR-6761-5p, miR-330-5p, miR-3127-5p, miR-99a-5p, miR-874-3p, miR-132-3p, miR-625-3p, and miR-195-3p were downregulated in COAD tissues. Furthermore, miR-155-5p, miR-582-5p, miR-452-3p, miR-146b-5p, miR-552-5p, miR-452-5p, miR-224-5p, miR-582-3p, and miR-592 were upregulated in COAD tissues (**Figures 6A–R**).

## Discussion

Along with the improvement of sequencing technology, the role of TMB as a biomarker for predicting the efficacy of ICIs has been confirmed in a number of clinical studies (32–34). TMB provides a quantitative estimation of the total number of mutations in the coding region of the tumor genome. The mutations were processed into neoantigens and presented to T cells through the major histocompatibility complex protein. To escape immune elimination, cancers use checkpoints that inhibit T cell response. ICIs change the treatment of cancer by reactivating T cells Therefore, tumor cells with higher levels of TMB may be more likely to be recognized by the immune system, which in turn triggers a stronger immune response to ICIs (35). There is a correlation between TMB and the benefits of combined immunotherapy for NSCLC (36). Previous studies have shown that blood-derived TMB detection does not require tissue samples, as only plasma samples are needed to evaluate TMB levels (37). Blood-derived TMB detection is an important substitute because biopsies for testing TMB cannot be obtained from many patients and the low invasiveness and high flexibility of using blood are additional advantages (37, 38). However, the evaluation of TMB using blood is challenging. For instance, the amount of DNA extracted from tumor cells circulating in the blood is often extremely small because it is a small fraction of free DNA. In recent years, a large number of studies have reported that miRNAs play an important role in the immune system through the differentiation and development of immune cells, the regulation of immune responses, and the occurrence and development of tumors (39–41). Therefore, a blood-based miRNA signature was established for a large portion of patients with advanced cancer who were unable to undergo surgery (42). However, in some special types of tumors, such as gastric cancer and colorectal cancer, although they cannot undergo surgery, tissue samples can be obtained using endoscopy.

The relationship between miRNA expression and TMB has not been previously reported in patients with COAD. In the present study, the differentially expressed miRNAs in samples with high and low TMB levels were identified as a signature, because they could effectively discriminate between high and low levels of TMB in the samples. This signifies that changes in genomics may affect the transcriptome to some extent. After selection of differentially expressed miRNAs, an 18-miRNA-based model was obtained from the training set, and along with the test set, it was used to verify the robustness and general applicability of the method. The accuracies in the training, test, and total sets were 0.9753, 0.964, and 0.9598, respectively. Analysis of the ROC curves demonstrated that the AUC in the training, test, and total set was 0.9984, 0.958, and 0.9817, respectively, which demonstrated that the model was highly credible. According to the SP and NPV in the training, test, and total set, respectively, the model effectively identified low TMB levels, as can be seen from the PPV. In addition, the model effectively identified high TMB levels.

Previous studies have suggested TMB as a potential independent biomarker and that it has a certain predictive value for the curative effect of treatments. A recent series of clinical studies concerning this problem confirmed the positive correlation between TMB values and the curative effect of treatments. In addition, the studies confirmed that the TMB value is independently related to the efficacy of immunotherapy but is not affected by the PD-L1 level, while the effect of immunotherapy on patients with a high TMB and PD-L1 levels was better. Non-synonymous mutations in tumor cells can lead to the production of new antigens, and the existence of tumor-specific new antigens is related to an increase in tumor immunogenicity. Consequently, the immune system can enhance the activity of T cells against tumors after recognizing a large number of new antigens, thus enhancing the efficacy of ICIs (43). Some studies have shown that if the tumor presents higher TMB levels and more new antigens, the tumor may respond better to immunotherapy (44, 45). In the present study, there were multiple differentially expressed miRNAs between tumors with different TMB levels, which were associated with immunity. Target gene enrichment analysis of miRNA in the model showed that these miRNAs are related to the process of transcriptome profiling and immunity. For example, the “DNA-binding transcription activator activity, RNA polymerase II-specific” and “nuclear hormone receptor binding” are involved in the process of transcriptome profiling. The “transforming growth factor beta receptor, cytoplasmic mediator activity” and “cell adhesion molecule binding”, etc. are related to the immune process. The target genes of these 18 miRNAs were also enriched in pathways in cancer, including “colorectal cancer.” Therefore, the results of the present study demonstrate that the 18-miRNA-based model is robust and functional in predicting TMB levels.

In the present study, we developed and verified a potential signature that predicts and discriminates between high and low TMB levels in patients with COAD. However, we detected some deficiencies in this signature. First, the threshold of discrimination between different TMB levels may vary from method to method. Second, a larger cohort of COAD patients should be used to improve the credibility of the signature. Third, the biological mechanism of these 18 miRNAs in tumor immunology requires further exploration. In addition, further experimental verification is recommended. According to the results of previous research, miRNAs circulating in the plasma are also associated with the immunotherapy of non-small cell lung cancer (46, 47).

## Conclusion

Based on the abovementioned reasons, we found that miRNA expression in COAD patients is different at different levels of TMB. An miRNA-based model was established as a signature to predict and discriminate between different TMB levels in patients with COAD.

## Data Availability Statement

The original contributions presented in the study are included in the article/supplementary material. Further inquiries can be directed to the corresponding authors.

## Ethics Statement

The studies involving human participants were reviewed and approved by The Affiliated Hospital of Qingdao university ethics committee. The patients/participants provided their written informed consent to participate in this study.

## Author Contributions

WX, YW, YX and CY performed the experiments, designed the research, analyzed the data, and wrote the manuscript. ZN and CZ provided reagents and intellectual guidance. ZG, CG, and CW analyzed the data, and wrote the manuscript. All authors contributed to the article and approved the submitted version.

## Funding

This work was supported by the National Natural Science Foundation of China (No.81600490 and 81600691), China Postdoctoral Science Foundation (2016M602098 and 2018M640615), the Research and Development Project of Shandong Province (No. 2016GSF201221), Young Taishan Scholars Program of Shandong Province (No.2019010668), and Qingdao Postdoctoral Science Foundation (2016046), Shandong Higher Education Young Science and Technology Support Program (grant number 2020KJL005).

## Conflict of Interest

The authors declare that the research was conducted in the absence of any commercial or financial relationships that could be construed as a potential conflict of interest.

## Abbreviations

SE, sensitivity; SP, specificity; PPV, positive predictive value; NPV, negative predictive value; ROC, receiver operating characteristic; AUC, area under the curve; LASSO, least absolute shrinkage and selection operator; PCA, principal component analysis; COAD, colon adenocarcinoma; TMB, Tumor mutation burden; PD-L1, programmed death protein-1(PD-1)/programmed death receptor ligand; ICIs, immune checkpoint inhibitors; miRNAs, microRNAs.
